# Thermodynamical Extension of a Symplectic Numerical Scheme with Half Space and Time Shifts Demonstrated on Rheological Waves in Solids

**DOI:** 10.3390/e22020155

**Published:** 2020-01-28

**Authors:** Tamás Fülöp, Róbert Kovács, Mátyás Szücs, Mohammad Fawaier

**Affiliations:** 1 Department of Energy Engineering, Faculty of Mechanical Engineering, BME, 1521 Budapest, Hungary; kovacsrobert@energia.bme.hu (R.K.); szucsmatyas@energia.bme.hu (M.S.); 2 Montavid Thermodynamic Research Group, 1112 Budapest, Hungary; 3 Department of Theoretical Physics, Wigner Research Centre for Physics, Institute for Particle and Nuclear Physics, 1525 Budapest, Hungary; 4 Department of Building Service and Process Engineering, Faculty of Mechanical Engineering, BME, 1521 Budapest, Hungary; fawaier@epget.bme.hu

**Keywords:** symplectic numerical methods, rheology, solids, waves

## Abstract

On the example of the Poynting–Thomson–Zener rheological model for solids, which exhibits both dissipation and wave propagation, with nonlinear dispersion relation, we introduce and investigate a finite difference numerical scheme. Our goal is to demonstrate its properties and to ease the computations in later applications for continuum thermodynamical problems. The key element is the positioning of the discretized quantities with shifts by half space and time steps with respect to each other. The arrangement is chosen according to the spacetime properties of the quantities and of the equations governing them. Numerical stability, dissipative error, and dispersive error are analyzed in detail. With the best settings found, the scheme is capable of making precise and fast predictions. Finally, the proposed scheme is compared to a commercial finite element software, COMSOL, which demonstrates essential differences even on the simplest—elastic—level of modeling.

## 1. Introduction

Numerical solution methods for dissipative problems are important and are a nontrivial topic. Already for reversible systems, the difference between a symplectic and nonsymplectic finite difference method is striking: the former can offer reliable prediction that stays near the exact solution even at extremely large time scales, while the latter may provide a solution that drifts away from the exact solution steadily. For dissipative systems, the situation is harder. Methods that were born with reversibility in mind may apparently fail for a nonreversible problem. For example, a finite element software is able to provide, at the expense of a large run time, a quantitatively and even a qualitatively wrong outcome, while a simple finite difference scheme solves the same problem fast and precisely [1].

Partly inspired by, and partly based on, the intensive development on symplectic schemes for reversible problems, remarkable research has been done in recent years to develop geometric methods for dissipative systems, more on ones with finite degrees of freedom (including [2,3,4,5,6,7]) and less for continua (see, e.g., [8,9,10,11]).

Thermodynamics also modifies the way of thinking concerning numerical modeling. Even if quantities known from mechanics form a closed system of equations to solve numerically, monitoring temperature (or other thermodynamical quantities) for a nonreversible system can give insight on the processes and phenomena, for example, pointing out the presence of viscoelasticity/rheology, and displaying when plastic changes start [12]. In addition, temperature can also react in the form of thermal expansion and heat conduction, even in situations where one is not prepared for this “surprise” [13].

Furthermore, in a sense, thermodynamics is a stability theory. Therefore, how thermodynamics ensures asymptotic stability for systems may give new ideas on how the stability and suppression of errors can be achieved for numerical methods. A conceptually closer relationship is desirable between these two areas.

Along these lines, we present a study where a new numerical scheme is suggested and applied for a continuum thermodynamical model. The scheme proves to be an extension of a symplectic method. In parallel, our finite difference scheme introduces a staggered arrangement of quantities by half space and time steps with respect to each other, according to the spacetime nature of the involved quantities and the nature of equations governing them. The shifts can be introduced by inspecting the equations. It turns out that balances, kinematic equations, and Onsagerian equations all have their own distinguished discretized realization. The shifts also render the scheme with one order of accuracy higher than the original symplectic scheme.

The continuum system that we take as the subject of our investigation is important on its own—it is the Poynting–Thomson–Zener (PTZ) rheological model for solids. This model exhibits both dissipation and wave propagation (actually, dispersive wave propagation), and this is thus ideal for testing various aspects and difficulties. Meanwhile, its predictions are relevant for many solids, typically ones with complicated micro- or mesoscopic structure, such as rocks [14,15,16], plastics [12], and asphalt. This non-Newtonian rheological model can explain why slow and fast measurements and processes give different results.

Solutions in the force-equilibrial and space-independent limit have proved successful in explaining experimental results [12]. Space-dependent—but still force-equilibrial—analytical solutions can model the opening of a tunnel, the gradual loading of thick-walled tubes and spherical tanks, and other problems [17]. The next level is to leave the force-equilibrial approximation, partly in order to cover and extend the force-equilibrial results but also to be utilized for evaluating measurements that include wave propagation as well. The present work is, in this sense, the next step in this direction.

## 2. Properties of the Continuum Model

The system that we consider is a homogeneous solid with Poynting–Thomson–Zener rheology, in small-strain approximation (hence, there is no need to distinguish between Lagrangian and Eulerian variables, or between material manifold vectors, covectors, tensors, etc. and spatial spacetime ones), in one space dimension (1D). Notably, the numerical scheme we introduce in the following section can be generalized to 2D and 3D with no difficulty (the results of our ongoing research on 2D and 3D are to be communicated later). For the present 1D treatment, we keep the technical details at a minimum so that we can focus on the key ideas.

The set of equations we discuss is, accordingly,

(1) $$\begin{array}{cc} \varrho \frac{\partial v}{\partial t} & =\frac{\partial \sigma }{\partial x} \end{array}$$

(2) $$\begin{array}{cc} \frac{\partial \varepsilon }{\partial t} & =\frac{\partial v}{\partial x} \end{array}$$

(3) $$\begin{array}{cc} \sigma +\tau \frac{\partial \sigma }{\partial t} & =E\varepsilon +\hat{E}\frac{\partial \varepsilon }{\partial t} \end{array}$$

where ϱ is mass density (constant in the small-strain approximation), (1) tells us how the spatial derivative of stress σ determines the time derivative of the velocity field *v* (volumetric force density being omitted for simplicity), (2) is the kinematic relationship between the strain field ε and *v*, and the rheological relationship (3) contains, in addition to Young’s modulus *E*, two positive coefficients $\hat{E},\tau$. (We note that, in the present context, ε can be used as the thermodynamical state variable for elasticity, but not in general; see [18,19]).

The PTZ model is a subfamily within the Kluitenberg–Verhás model family, which can be obtained via a nonequilibrium thermodynamical internal variable approach [20]. The PTZ model looks particularly simple, after eliminating the internal variable, both in specific energy $e_{\mathrm{total}}$ and in specific entropy *s*:

(4) $$\begin{array}{cc} e_{\mathrm{total}} & =e_{\mathrm{kinetic}}+e_{\mathrm{thermal}}+e_{\mathrm{elastic}}+e_{\mathrm{rheological}}\equiv \frac{1}{2}v^{2}+c_{\sigma }T+\frac{E}{2\varrho }\varepsilon^{2}+\frac{\tau }{2\varrho \hat{I}}{\left(\sigma -E\varepsilon \right)}^{2} \end{array}$$

(5) $$\begin{array}{cc} s & =c_{\sigma }\ln\frac{T}{T_{\mathrm{aux}}} \end{array}$$

where thermal expansion and heat conduction are neglected, and the “isobaric” specific heat $c_{\sigma }$ is assumed constant for simplicity (a)long the lines of [20], Appendix B. *T* is the absolute temperature, the auxiliary constant $T_{\mathrm{aux}}$ is present on dimensional grounds, and the “index of damping” $\hat{I}$ [20] is

(6) $$\begin{array}{c} \hat{I}=\hat{E}-\tau E>0, \end{array}$$

the inequality being a consequence of the second law of thermodynamics. Moreover, in this simple setting, the entropy production rate density

(7) $$\begin{array}{c} \frac{1}{T}\frac{{\left(\sigma -E\varepsilon \right)}^{2}}{\hat{I}} \end{array}$$

increases temperature directly:

(8) $$\begin{array}{c} \varrho c_{\sigma }\frac{T}{t}=\frac{{\left(\sigma -E\varepsilon \right)}^{2}}{\hat{I}}. \end{array}$$

Equation (8) can be derived by taking ϱT times the time derivative of (5), together with the balance of entropy and the fact that, with neglected heat conduction, entropy current density has also been set to zero.

Remarkably, also thanks to our simplifications, the closed system of Equations (1)–(3) to be solved is linear. Having the solution for *v*, ε, and σ, the further quantities (*T*, *s*, and the various energy terms) can be obtained.

Our system admits two distinguished time scales, τ and

(9) $$\begin{array}{c} \hat{\tau }=\frac{\hat{E}}{E}>\tau , \end{array}$$

the inequality following from (6). For phenomena much slower than these time scales, the rule-of-thumb approximation of keeping only the lowest time derivative for any quantity present in (3) gives the Hooke model

(10) $$\begin{array}{c} \sigma =E\varepsilon , \end{array}$$

formally the τ→0, $\hat{E}\to 0$ ($\hat{\tau }\to 0$) limit of (3). The system of Equations (1) and (2), (10) leads to a wave equation for *v*, σ, ε each, with wave speed

(11) $$\begin{array}{c} c=\sqrt{\frac{E}{\varrho }}. \end{array}$$

On the other side, for processes much faster then the two time scales, keeping the highest time derivatives leads to

(12) $$\begin{array}{cccccccc} \tau \frac{\partial \sigma }{\partial t} & =\hat{E}\frac{\partial \varepsilon }{\partial t},\; & \frac{\partial \sigma }{\partial t} & =\frac{\hat{E}}{\tau }\frac{\partial \varepsilon }{\partial t},\; & \; & ⟹\;\int_{t_{1}}^{t_{2}}dt\;\;\mathrm{gives} & \Delta_{t_{1}\to t_{2}}^{}\sigma & =\frac{\hat{E}}{\tau }\Delta_{t_{1}\to t_{2}}^{}\varepsilon ; \end{array}$$

that is, for stress and strain changes (e.g., for deviations from initial values), the system effectively behaves like a Hooke model, with a “dynamic” Young’s modulus

(13) $$\begin{array}{c} E_{\infty }=\frac{\hat{E}}{\tau },\;\;E_{\infty }>E. \end{array}$$

The corresponding effective wave equation possesses the wave speed

(14) $$\begin{array}{c} \hat{c}=\sqrt{\frac{E_{\infty }}{\varrho }}=\sqrt{\frac{\hat{E}}{\tau \varrho }},\;\;\hat{c}>c. \end{array}$$

For a more rigorous and closer investigation of these aspects, the dispersion relation can be derived. Namely, on the line −∞<x<∞, any (not too pathological) field can be given as a continuous linear combination of $e^{ikx}$ space dependences, where the “wave number” *k* is any real parameter. If such a (Fourier) decomposition is done at, say, t=0, then the subsequent time dependence of one such mode may be particularly simple:

(15) $$\begin{array}{c} \left(\begin{array}{c} v\\ \varepsilon \\ \sigma \end{array}\right)\left(0,x\right)=\left(\begin{array}{c} iA_{v}\left(k\right)\\ A_{\varepsilon }\left(k\right)\\ A_{\sigma }\left(k\right) \end{array}\right)e^{ikx},\;\;\left(\begin{array}{c} v\\ \varepsilon \\ \sigma \end{array}\right)\left(t,x\right)=\left(\begin{array}{c} iA_{v}\left(k\right)\\ A_{\varepsilon }\left(k\right)\\ A_{\sigma }\left(k\right) \end{array}\right)e^{-i\omega t}e^{ikx} \end{array}$$

with some appropriate ω, which is complex, in general; the factor i in the first component is introduced in order to be in tune with later convenience. A space and time dependence

(16) e−iωteikx=eImωte−iReωteikx=e−(−Imω)teikx−Reωkt

expresses traveling with a constant velocity Reωk and an exponential decrease (for dissipative systems like ours, Imω<0). In general, how many ω variables are possible depends on the number of fields and on the order of time derivatives. In our case, deriving the relationship between compatible ω and *k*—the dispersion relation—is straightforward:

(17) $$\begin{array}{c} \omega^{2}\frac{1-i\tau \omega }{1-i\hat{\tau }\omega }=c^{2}k^{2}. \end{array}$$

In the limit $\left|\omega \right|\to 0$ (limit of slow processes), we find

(18) ω2=c2k2,ω=±ck,Reωk=ωk=±c,

while in the opposite limit $\left|\omega \right|\to \infty$ (limit of fast processes), the result is

(19) ω2−iτω−iτ^ω=ω2ττ^=c2k2,ω=±c^k,Reωk=ωk=±c^.

Both results confirm the findings above ((11) and (14), respectively).

This is a point where we can see the importance of the PTZ model. Namely, when measuring Young’s modulus (or, in 3D, the two elasticity coefficients) of a solid, the speed of uniaxial loading or the frequency of sound in an acoustic measurement may influence the outcome, and an adequate/sufficient interpretation may come in terms of a PTZ model. Indeed, in rock mechanics, dynamic elastic moduli are long known to be larger than their static counterparts (a new and comprehensive study on this, [21], is in preparation), in accordance with the thermodynamics-originated inequality in (14) (or its 3D version).

## 3. The Numerical Scheme

The classic attitude to finite difference schemes is that all quantities are registered at the same discrete positions and at the same discrete instants. An argument against this practice is that, when dividing a sample into finite pieces, some physical quantities have a meaning related to the bulk, the center of a piece, while others have a physical role related to the boundaries of a unit. For example, in Fourier heat conduction, heat flux is proportional to the gradient of temperature. A natural discretization of this, in one space dimension, is that temperature values sit at the centers and heat flux values at the boundaries—in other words, at a half space step distance from the centers [1]. In addition, in heat conduction, the change rate of specific internal energy is determined by the divergence of the heat flux. The natural one space dimensional discretization is then that, since heat flux values sit at the boundaries, specific internal energy values $e_{\mathrm{thermal}}$ are placed at the centers (at the same places where temperature values *T* are put, which is in tune with the fact that the two are related to one another via $e_{\mathrm{thermal}}=c_{\sigma }T$) [1]. More generally, in continuum theories, specific extensive and density quantities would naturally live at a center, while currents/fluxes are boundary-related by their physical nature/role.

Here, we generalize this approach. Namely, when one has a full—at the general level, 4D—spacetime perspective (which unfolds that traditional physical quantities are time- and spacelike components of four-vectors, four-covectors, four-cotensors etc. governed by 4D equations with four-divergences, four-gradients etc.), then it turns out that quantities may “wish” to be staggered with respect to each other by a half in time as well. Taking again the example of the balance of internal energy in heat conduction, the finite-difference discretization of the change rate of specific internal energy $e_{\mathrm{thermal}}$ contains the change $\Delta e_{\mathrm{thermal}}$ corresponding to a finite time difference Δt. This change is caused by the flux of heat leaving the spatial unit during this time interval Δt, which is the time average of the flux naturally realized at half-time Δt/2. Accordingly, heat flux values would be realized as half-shifted in time with respect to specific internal energy.

More generally, if an equation relates the change rate of a quantity to another quantity, then these two quantities would be realized as half-shifted in time with respect to one another.

To sum up, the space and time derivatives suggest how we can arrange the quantities with space and time half-shifts, respectively.

This approach is what we realize for the present system. Discrete space and time values are chosen as

(20) $$\begin{array}{c} x_{n}=n\Delta x,\;n=0,1,\ldots ,N,\;t^{j}=j\Delta t,\;j=0,1,\ldots ,J, \end{array}$$

and discrete values of stress are prescribed to these spatial and temporal coordinates:

(21) $$\begin{array}{c} \sigma_{n}^{j}\;\mathrm{at}\;t^{j},\;x_{n}. \end{array}$$

Next, investigating (1), we decide to put velocity values half-shifted with respect to stress values both in space and time:

(22) $$\begin{array}{c} v_{n+1/2}^{j-1/2}\;\mathrm{at}\;t^{j}-\frac{\Delta t}{2},\;x_{n}+\frac{\Delta x}{2}, \end{array}$$

and discretize (1) as

(23) $$\begin{array}{cc} \varrho \frac{v_{n+1/2}^{j+1/2}-v_{n+1/2}^{j-1/2}}{\Delta t}\; & =\frac{\sigma_{n+1}^{j}-\sigma_{n}^{j}}{\Delta x}. \end{array}$$

Next, studying (2) suggests analogously that we should have strain values half-shifted with respect to velocity values both in time and space. Therefore, strain is to reside at the same spacetime location as stress:

(24) $$\begin{array}{c} \varepsilon_{n}^{j}\;\mathrm{at}\;t^{j},\;x_{n}, \end{array}$$

and (2) is discretized as

(25) $$\begin{array}{cc} \frac{\varepsilon_{n}^{j+1}-\varepsilon_{n}^{j}}{\Delta t}\; & =\frac{v_{n+1/2}^{j+1/2}-v_{n-1/2}^{j+1/2}}{\Delta x}. \end{array}$$

Finally, for the Hooke model, (10) is discretized plainly as

(26) $$\begin{array}{c} \sigma_{n}^{j}=E\varepsilon_{n}^{j}, \end{array}$$

as stress and strain are assigned to the same locations. In the Hooke case, bookkeeping both stress and strain is redundant.

**Figure 1:**
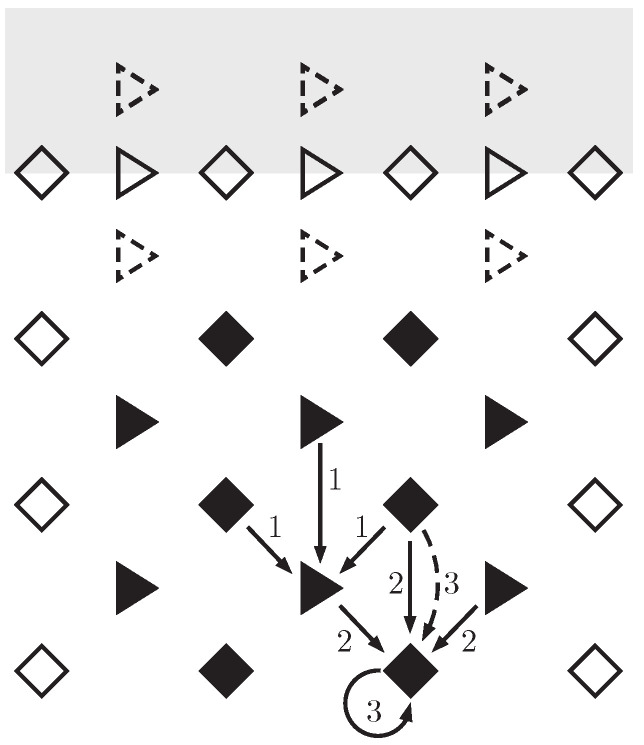
Visualization of the finite difference numerical scheme. Velocity values stay at triangles, strain and stress values at rhombuses, and filled symbols denote values calculated via the scheme, while empty ones represent initial and boundary conditions. First, new velocities are determined from (23), new strains are obtained according to (25), and new stress values are obtained from (26) or (32). Grey indicates initial condition values (which are typically known for a whole time interval in practice). If the “grey dashed triangles” are not available, then an explicit Euler step can be used to produce the “white dashed triangles” for starting the scheme.

Rewriting the scheme for the Hooke case as

(27) $$\begin{array}{cccc} v_{n+1/2}^{j+1/2} & =v_{n+1/2}^{j-1/2}+\frac{E}{\varrho }\frac{\Delta t}{\Delta x}\left(\varepsilon_{n+1}^{j}-\varepsilon_{n}^{j}\right),\; & \varepsilon_{n}^{j+1} & =\varepsilon_{n}^{j}+\frac{\Delta t}{\Delta x}\left(v_{n+1/2}^{j+1/2}-v_{n-1/2}^{j+1/2}\right),\; \end{array}$$

we can recognize the steps of the symplectic Euler method [22] (with the Hamiltonian corresponding to $e_{\mathrm{kinetic}}+e_{\mathrm{elastic}}$). Now, a symplectic method is highly favorable because of its extremely good large-time behavior, including the preservation of energy conservation. While (27) coincides with the symplectic Euler method computationally, the present interpretation of the quantities is different, because of the space and time staggering. One advantageous consequence is that, due to the reflection symmetries (see Figure 1), our scheme makes second-order accurate predictions (understood in powers of Δt and Δx), while the symplectic Euler method makes only first-order accurate ones [22]. Indeed, setting

(28) $$\begin{array}{c} t=t^{j},\;x=x_{n}+\frac{\Delta x}{2} \end{array}$$

and assuming that

(29) $$\begin{array}{cccccc} v_{n+1/2}^{j-1/2} & =v\left(t-\frac{\Delta t}{2},x\right), & \varepsilon_{n+1}^{j} & =\varepsilon \left(t,x+\frac{\Delta x}{2}\right), & \varepsilon_{n}^{j} & =\varepsilon \left(t,x-\frac{\Delta x}{2}\right) \end{array}$$

exactly, the error of the prediction for $v_{n}^{j+1}$ is

(30) $$\begin{array}{cc} v_{n+1/2}^{j+1/2}-v\left(t+\frac{\Delta t}{2},x\right) & =v_{n+1/2}^{j-1/2}+\frac{E}{\varrho }\frac{\Delta t}{\Delta x}\left(\varepsilon_{n+1}^{j}-\varepsilon_{n}^{j}\right)-v\left(t+\frac{\Delta t}{2},x\right)\\ \; & =v\left(t-\frac{\Delta t}{2},x\right)-v\left(t+\frac{\Delta t}{2},x\right)+\frac{E}{\varrho }\frac{\Delta t}{\Delta x}\left[\varepsilon \left(t,x+\frac{\Delta x}{2}\right)-\varepsilon \left(t,x-\frac{\Delta x}{2}\right)\right]\\ \; & =-\frac{\partial v}{\partial t}\left(t,x\right)\Delta t+O\left(\Delta t^{3}\right)+\frac{E}{\varrho }\frac{\Delta t}{\Delta x}\left[\frac{\partial \varepsilon }{\partial x}\left(t,x\right)\Delta x+O\left(\Delta x^{3}\right)\right]\\ \; & =O\left(\Delta t^{3}\right)+O\left(\Delta t\Delta x^{2}\right) \end{array}$$

after Taylor series expansion, cancellations, and the use of (1).

Analogously, with

(31) $$\begin{array}{c} t=t^{j}+\frac{\Delta t}{2},\;x=x_{n}, \end{array}$$

the second-order accuracy of prediction $\varepsilon_{n}^{j+1}$ can be proved.

In case of the PTZ model, we need to discretize (3). Here, both σ and its derivative, and both ε and its derivative, appear. Hence, staggering does not directly help us. This is what one can expect for dissipative, irreversible, relaxation-type equations in general. However, an interpolation-like solution is possible:

(32) $$\begin{array}{cc} \alpha \sigma_{n}^{j}+\left(1-\alpha \right)\sigma_{n}^{j+1}+\tau \frac{\sigma_{n}^{j+1}-\sigma_{n}^{j}}{\Delta t} & =E\left[\alpha \varepsilon_{n}^{j}+\left(1-\alpha \right)\varepsilon_{n}^{j+1}\right]+\hat{E}\frac{\varepsilon_{n}^{j+1}-\varepsilon_{n}^{j}}{\Delta t} \end{array}$$

where α=1/2 is expected to provide second-order accurate prediction, and other seminal values are α=1 (the explicit case, which is expected to be stiff) and α=0 (the fully implicit case).

For a generic α, (32) looks implicit. However, thermodynamics has brought in an *ordinary* differential equation-type extension to the Hooke continuum, not a *partial* one, and a linear one, in fact. Thus, (32) can be rewritten in explicit form:

(33) $$\begin{array}{c} \sigma_{n}^{j+1}=\frac{1}{1-\alpha +\frac{\tau }{\Delta t}}\left\{\left(\frac{\tau }{\Delta t}-\alpha \right)\sigma_{n}^{j}+E\left[\alpha \varepsilon_{n}^{j}+\left(1-\alpha \right)\varepsilon_{n}^{j+1}\right]+\hat{E}\frac{\varepsilon_{n}^{j+1}-\varepsilon_{n}^{j}}{\Delta t}\right\} \end{array}$$

assuming

(34) $$\begin{array}{c} 1-\alpha +\frac{\tau }{\Delta t}\neq 0. \end{array}$$

Verifying the second-order accuracy of (33) for α=1/2 is then straightforward, in complete analogy to the two previous proofs.

## 4. Stability

One may specify a space step Δx according to the given need, adjusted to the desirable spatial resolution. In parallel, the time step Δt is reasonably chosen to be considerably smaller than the involved time scales (e.g., τ and $\hat{\tau }$ of our example system). Now, a finite difference scheme may prove to be unstable for the taken Δx and Δt, making numerical errors (which are generated unavoidably because of floating-point round-off) increase essentially exponentially and ruining the usefulness of what we have done. Therefore, first, a stability analysis is recommended, to explore the region of good pairs of Δx, Δt for the given scheme and system.

We continue with this step for our scheme and system, performing a von Neumann investigation [23], where the idea is similar to the derivation of the dispersion relation. There, the time evolution of continuum Fourier modes was studied (see (15)), while here we examine whether errors, expanded in modes with $e^{ikx_{n}}$ space dependence, increase or not, during an iteration by one time step. For such linear situations as ours, when the iteration step means a multiplication by a matrix, such a mode may simply obtain a growth factor ξ (that is *k*-dependent but space-independent); in other words, the iteration matrix (frequently called the “transfer matrix”) has these modes as eigenvectors with the corresponding eigenvalues ξ. Thus, $\left|\xi \right|<1$ (for all *k*) ensures stability. Furthermore, $\left|\xi \right|=1$ means stability if the algebraic multiplicity of ξ—its multiplicity as a root of the characteristic polynomial of the transfer matrix—equals its geometric multiplicity—the number of linearly independent eigenvectors (eigenmodes), i.e., the dimension of the eigensubspace ([24], page 186, Theorem 4.13; [25], page 381, Proposition 2).

We find it important to emphasize the following. The stability of the corresponding physical model, the Poynting–Thomson–Zener body, is ensured by the second law of thermodynamics. Thus, asymptotic stability of the solutions is guaranteed. The numerical method, and thus the stability analysis, must reflect the thermodynamical (physical) requirements as well, together with the particular conditions related to the applied discretization method. In overall, these aspects are not independent of each other. Such a way of thinking is also emphasized in [26], in which a numerical method is developed for electrodynamical problems using staggered fields and expecting similar properties as in our case.

With boundary conditions specified, one can say more. (Actually, all continuum problems require boundary or asymptotic conditions—we also specify some in the forthcoming section on applications.) Boundary conditions may allow only certain combinations of $e^{ikx_{n}}$ as eigenmodes of the transfer matrix. Consequently, this type of analysis is more involved and is, therefore, usually omitted. As a general rule of thumb, one can expect that $\left|\xi \right|>1$ for some $e^{ikx_{n}}$ indicates instability for modes obeying the boundary conditions, while $\left|\xi \right|\leq 1$ for all $e^{ikx_{n}}$ suggests stability for all modes allowed by the boundary conditions. (Namely, the problem of differing multiplicities for $\left|\xi \right|=1$ can be wiped out by the boundary conditions.)

### 4.1. The Hooke Case

In the Hooke case, the “plane wave modes” for the two bookkept quantities *v*, ε can, for later convenience, be written as

(35) $$\begin{array}{ccccc} v_{n+1/2}^{j-1/2} & =iA_{v}^{j}e^{ik\left(n+\frac{1}{2}\right)\Delta x}, & \varepsilon_{n}^{j} & =A_{\varepsilon }^{j}e^{ikn\Delta x}, & k\Delta x\in [0,2\pi ), \end{array}$$

and the condition on *k* related to that *k* outside such a “Brillouin zone” makes the description redundant.

Realizing the iteration steps (27) as matrix products leads, for the amplitudes introduced in (35), to

(36) $$\begin{array}{cc} \left(\begin{array}{c} A_{v}^{j+1}\\ A_{\varepsilon }^{j+1} \end{array}\right) & =\left(\begin{array}{cc} 1 & 0\\ -2\frac{\Delta t}{\Delta x}S & 1 \end{array}\right)\cdot \left(\begin{array}{c} A_{v}^{j+1}\\ A_{\varepsilon }^{j} \end{array}\right)=\left(\begin{array}{cc} 1 & 0\\ -2\frac{\Delta t}{\Delta x}S & 1 \end{array}\right)\cdot \left(\begin{array}{cc} 1 & 2c^{2}\frac{\Delta t}{\Delta x}S\\ 0 & 1 \end{array}\right)\cdot \left(\begin{array}{c} A_{v}^{j}\\ A_{\varepsilon }^{j} \end{array}\right)\\ \; & =\left(\begin{array}{cc} 1 & 2c^{2}\frac{\Delta t}{\Delta x}S\\ -2\frac{\Delta t}{\Delta x}S & 1-4c^{2}\frac{\Delta t^{2}}{\Delta x^{2}}S^{2} \end{array}\right)\cdot \left(\begin{array}{c} A_{v}^{j}\\ A_{\varepsilon }^{j} \end{array}\right)\equiv T\cdot \left(\begin{array}{c} A_{v}^{j}\\ A_{\varepsilon }^{j} \end{array}\right) \end{array}$$

with

(37) $$\begin{array}{c} S=\sin\frac{k\Delta x}{2},\;0\leq S\leq 1. \end{array}$$

For space dependences (35),

(38) $$\begin{array}{c} v_{n+1/2}^{j+1/2}=\xi v_{n+1/2}^{j-1/2},\;\varepsilon_{n}^{j+1}=\xi \varepsilon_{n}^{j}\;\mathrm{lead}\;\mathrm{to}\;A_{v}^{j+1}=\xi A_{v}^{j},\;A_{\varepsilon }^{j+1}=\xi A_{\varepsilon }^{j}, \end{array}$$

in other words, to the eigenvalue problem

(39) $$\begin{array}{c} Ty=\xi y\;\mathrm{with}\;y=\left(\begin{array}{c} A_{v}^{j}\\ A_{\varepsilon }^{j} \end{array}\right). \end{array}$$

Let us introduce the notation

(40) $$\begin{array}{c} C=c\frac{\Delta t}{\Delta x} \end{array}$$

for the Courant number of our scheme for the Hooke system. Comparing the characteristic polynomial of T,

(41) $$\begin{array}{c} P\left(\xi \right)=\xi^{2}+\left(4C^{2}S^{2}-2\right)\xi +1 \end{array}$$

with its form written via its roots,

(42) $$\begin{array}{c} \left(\xi -\xi_{+}\right)\left(\xi -\xi_{-}\right)=\xi^{2}-\left(\xi_{+}+\xi_{-}\right)\xi +\xi_{+}\xi_{-}, \end{array}$$

reveals, on one side, that, in order to have both $\left|\xi_{+}\right|\leq 1$ and $\left|\xi_{+}\right|\leq 1$, both magnitudes have to be 1 (since their product is 1), which, on the other side, also implies

(43) $$\begin{array}{c} 4C^{2}S^{2}-2=-\xi_{+}-\xi_{-}\leq \left|\xi_{+}\right|+\left|\xi_{-}\right|\leq 2\;⟹\;C^{2}S^{2}\leq 1,\;CS\leq 1, \end{array}$$

as both *C* and *S* are non-negative.

If CS<1, then the two roots,

(44) $$\begin{array}{c} \xi_{\pm }=1-2C^{2}S^{2}\pm \sqrt{4C^{2}S^{2}\left(C^{2}S^{2}-1\right)}, \end{array}$$

are complex, with a unit modulus, and are the complex conjugate of one another. Especially simple—and principally distinguished, as we see in the next sections—is the case C=1. Thus,

(45) $$\begin{array}{c} \xi_{\pm }=e^{\pm ik\Delta x}, \end{array}$$

with the remarkable property that $\arg\xi_{\pm }$ linearly depends on *k*—which is to say that both branches of the discrete dispersion relation are linear.

In parallel, if CS=1, then the two roots coincide, $\xi_{\pm }=-1$. The algebraic multiplicity 2 is accompanied with geometric multiplicity 1: only the multiples of

(46) $$\begin{array}{c} y=\left(\begin{array}{c} c\\ -1 \end{array}\right) \end{array}$$

are eigenvectors. If C=1, then this affects only one mode, S=1, $k=\frac{\pi }{k}$, and if that mode is prohibited by the boundary conditions, then the choice C=1 ensures a stable scheme.

With C>1, CS≤1 would be violated by a whole interval for *k* (recall (37)), which may not be cured by boundary conditions, so the best candidate (the largest Δt for a fixed Δx, or the smallest possible Δx for fixed Δt) to have stability is C=1.

### 4.2. Poynting–Thomson–Zener Case

For the PTZ system, the von Neumann stability analysis of our discretization studies the modes

(47) $$\begin{array}{cccccc} v_{n+1/2}^{j-1/2} & =iA_{v}^{j}e^{ik\left(n+\frac{1}{2}\right)\Delta x}, & \varepsilon_{n}^{j} & =A_{\varepsilon }^{j}e^{ikn\Delta x}, & \sigma_{n}^{j} & =A_{\sigma }^{j}e^{ikn\Delta x}, \end{array}$$

on which iteration via

(48) $$\begin{array}{cc} v_{n+1/2}^{j+1/2} & =v_{n+1/2}^{j-1/2}+\frac{1}{\varrho }\frac{\Delta t}{\Delta x}\left(\sigma_{n+1}^{j}-\sigma_{n}^{j}\right),\;\;\;\varepsilon_{n}^{j+1}=\varepsilon_{n}^{j}+\frac{\Delta t}{\Delta x}\left(v_{n+1/2}^{j+1/2}-v_{n-1/2}^{j+1/2}\right), \end{array}$$

(49) $$\begin{array}{cc} \sigma_{n}^{j+1} & =\frac{1}{1-\alpha +\frac{\tau }{\Delta t}}\left\{\left(\frac{\tau }{\Delta t}-\alpha \right)\sigma_{n}^{j}+E\left[\alpha \varepsilon_{n}^{j}+\left(1-\alpha \right)\varepsilon_{n}^{j+1}\right]+\hat{E}\frac{\varepsilon_{n}^{j+1}-\varepsilon_{n}^{j}}{\Delta t}\right\}, \end{array}$$

gives

(50) $$\begin{array}{cc} \left(\begin{array}{c} A_{v}^{j+1}\\ A_{\varepsilon }^{j+1}\\ A_{\sigma }^{j+1} \end{array}\right)\; & =\left(\begin{array}{ccc} 1 & 0 & 0\\ 0 & 1 & 0\\ 0 & \frac{E\left(1-\alpha \right)+\frac{\hat{E}}{\Delta t}}{\left(1-\alpha \right)+\frac{\tau }{\Delta t}} & \frac{\tau \Delta t-\alpha }{\left(1-\alpha \right)+\frac{\tau }{\Delta t}} \end{array}\right)\cdot \left(\begin{array}{ccc} 1 & 0 & 0\\ -2\frac{\Delta t}{\Delta x}S & 1 & 0\\ 0 & 0 & 1 \end{array}\right)\cdot \left(\begin{array}{ccc} 1 & 0 & 2\frac{\Delta t}{\varrho \Delta x}S\\ 0 & 1 & 0\\ 0 & 0 & 1 \end{array}\right)\cdot \left(\begin{array}{c} A_{v}^{j}\\ A_{\varepsilon }^{j}\\ A_{\sigma }^{j} \end{array}\right)\\ \; & \;+\left(\begin{array}{ccc} 0 & 0 & 0\\ 0 & 0 & 0\\ 0 & \frac{E\alpha -\frac{\hat{E}}{\Delta t}}{\left(1-\alpha \right)+\frac{\tau }{\Delta t}} & 0 \end{array}\right)\cdot \left(\begin{array}{c} A_{v}^{j}\\ A_{\varepsilon }^{j}\\ A_{\sigma }^{j} \end{array}\right)\equiv \hat{T}\left(\begin{array}{c} A_{v}^{j}\\ A_{\varepsilon }^{j}\\ A_{\sigma }^{j} \end{array}\right) \end{array}$$

with

(51) $$\begin{array}{c} \begin{array}{c} \hat{T}=\left(\begin{array}{ccc} 1 & 0 & 2\frac{\Delta t}{\varrho \Delta x}S\\ -2\frac{\Delta t}{\Delta x}S & 1 & -4\frac{\Delta t^{2}}{\varrho \Delta x^{2}}S^{2}\\ -2\frac{E\left(1-\alpha \right)+\frac{\hat{E}}{\Delta t}}{\left(1-\alpha \right)+\frac{\tau }{\Delta t}}\cdot \frac{\Delta t}{\Delta x}S & \frac{E}{\left(1-\alpha \right)+\frac{\tau }{\Delta t}} & \frac{\frac{\tau }{\Delta t}-\alpha }{\left(1-\alpha \right)+\frac{\tau }{\Delta t}}-4\frac{E\left(1-\alpha \right)+\frac{\hat{E}}{\Delta t}}{\left(1-\alpha \right)+\frac{\tau }{\Delta t}}\cdot \frac{\Delta t^{2}}{\varrho \Delta x^{2}}S^{2} \end{array}\right). \end{array} \end{array}$$

The characteristic polynomial is now

(52) $$\begin{array}{c} \hat{P}\left(\xi \right)=a_{3}\xi^{3}+a_{2}\xi^{2}+a_{1}\xi +a_{0}, \end{array}$$

(53) $$\begin{array}{cccccccc} a_{0} & =\frac{\alpha -\frac{\tau }{\Delta t}}{\left(1-\alpha \right)+\frac{\tau }{\Delta t}},\; & a_{1} & =3-\frac{2-4\left(\alpha -\frac{\hat{\tau }}{\Delta t}\right)C^{2}S^{2}}{\left(1-\alpha \right)+\frac{\tau }{\Delta t}},\; & a_{2} & =-3+\frac{1+4\left[\left(1-\alpha \right)+\frac{\hat{\tau }}{\Delta t}\right]C^{2}S^{2}}{\left(1-\alpha \right)+\frac{\tau }{\Delta t}},\; & a_{3} & =1. \end{array}$$

Three roots are considerably more difficult to directly analyze. One alternative is to use Jury’s criteria [27] for whether the roots are within the unit circle of the complex plane, and another possibility is to apply the Möbius transformation $\xi =\frac{\eta +1}{\eta -1}$ on (52) and utilize the Routh–Hurwitz criteria to determine whether the mapped roots are within the left half plane. The two approaches provide the same result. Nevertheless, one criterion provided by one of these two methods may not directly be one criterion of the other method. It is only the combined result (the intersection of the conditions) that agrees. Accordingly, it can be beneficial to perform both investigations because it may be laboring to recognize a simple condition provided by one of the routes as a consequence of the conditions directly offered by the other route.

Jury’s criteria, for our case, are as follows. First, $\hat{P}\left(1\right)>0$ gives

(54) $$\begin{array}{c} \frac{4C^{2}S^{2}}{\left(1-\alpha \right)+\frac{\tau }{\Delta t}}>0,\;⟺\;\left(1-\alpha \right)+\frac{\tau }{\Delta t}>0. \end{array}$$

Second, ${\left(-1\right)}^{3}\hat{P}\left(-1\right)>0$ yields

(55) $$\begin{array}{c} 8-8\frac{\frac{1}{2}+\left[\left(\frac{1}{2}-\alpha \right)+\frac{\hat{\tau }}{\Delta t}\right]C^{2}S^{2}}{\left(1-\alpha \right)+\frac{\tau }{\Delta t}}>0, \end{array}$$

which, in light of (54), reduces to

(56) $$\begin{array}{c} \left(\frac{1}{2}-\alpha \right)+\frac{\tau }{\Delta t}>\left[\left(\frac{1}{2}-\alpha \right)+\frac{\hat{\tau }}{\Delta t}\right]C^{2}S^{2}. \end{array}$$

Third, the matrices $\left(\begin{array}{cc} a_{3} & a_{2}\\ 0 & a_{3} \end{array}\right)\pm \left(\begin{array}{cc} 0 & a_{0}\\ a_{0} & a_{1} \end{array}\right)$ have to be positive innerwise. Following Jury [27], a matrix is *positive innerwise* if the determinant of the matrix and its all inners are positive. Here, the *inner* $\Delta_{m-2}$ of an m×m matrix is formed by deleting its first and *m*th rows and columns. Inner $\Delta_{m-4}$ is the inner of $\Delta_{m-2}$, and the procedure is continued until $\Delta_{1}$ or $\Delta_{2}$ is reached. Inners enter the picture only for m≥3, so, in our case, only positive definiteness of the matrices themselves is to be ensured. Now, the ‘+’ branch leads to

(57) $$\begin{array}{c} \left(\frac{1}{2}-\alpha \right)+\frac{\tau }{\Delta t}>\frac{\hat{\tau }-\tau }{\Delta t}C^{2}S^{2}, \end{array}$$

which is weaker than (56), because there the rhs is larger by $\left[\left(1-\alpha \right)+\frac{\tau }{\Delta t}\right]C^{2}S^{2}$ (and cf. (54)). Meanwhile, the ‘−’ branch induces condition

(58) $$\begin{array}{c} \hat{\tau }>\tau , \end{array}$$

which we have already encountered in (9) as the thermodynamical requirement (6) at the continuum level, and which also induces, via (57),

(59) $$\begin{array}{c} \left(\frac{1}{2}-\alpha \right)+\frac{\tau }{\Delta t}>0, \end{array}$$

which is stronger than (54). This also allows us to rearrange (56) and exploit it as

(60) $$\begin{array}{c} C^{2}S^{2}<\frac{\left(\frac{1}{2}-\alpha \right)+\frac{\tau }{\Delta t}}{\left(\frac{1}{2}-\alpha \right)+\frac{\hat{\tau }}{\Delta t}}<1\;\mathrm{for}\;\mathrm{all}\;0\leq S\leq 1\;⟹\;C^{2}<\frac{\left(\frac{1}{2}-\alpha \right)+\frac{\tau }{\Delta t}}{\left(\frac{1}{2}-\alpha \right)+\frac{\hat{\tau }}{\Delta t}}<1. \end{array}$$

Conditions (58)–(60) summarize the obtained stability requirements, the first referring to the constants of the continuum model only, the second relating α and Δt of the scheme, and the third limiting Δx (through *C*) in light of α and Δt.

If, instead of Jury’s criteria, one follows the Routh–Hurwitz path on the Möbius transformed polynomial,

(61) $$\begin{array}{c} \hat{Q}\left(\eta \right)={\left(\eta -1\right)}^{3}\hat{P}\left(\frac{\eta +1}{\eta -1}\right)=b_{3}\eta^{3}+b_{2}\eta^{2}+b_{1}\eta +b_{0}, \end{array}$$

(62) $$\begin{array}{cccc} b_{0} & =8\varrho \Delta x^{2}\left\{\left[\left(\frac{1}{2}-\alpha \right)+\frac{\tau }{\Delta t}\right]-\left[\left(\frac{1}{2}-\alpha \right)+\frac{\hat{\tau }}{\Delta t}\right]C^{2}S^{2}\right\},\; & b_{1} & =4\varrho \Delta x^{2}\left(1-C^{2}S^{2}\right)\Delta t, \end{array}$$

(63) $$\begin{array}{cccc} b_{2} & =8\left[\left(\frac{1}{2}-\alpha \right)+\frac{\tau }{\Delta t}\right]E\Delta t^{2}S^{2},\; & b_{3} & =4E\Delta t^{3}S^{2}, \end{array}$$

then, having $b_{3}>0$, roots lie in the left half plane if all corner subdeterminants of $\left(\begin{array}{ccc} b_{2} & b_{0} & 0\\ b_{3} & b_{1} & 0\\ 0 & b_{2} & b_{0} \end{array}\right)$ are positive; i.e., $b_{2}>0$, $b_{1}b_{2}-b_{0}b_{3}>0$ and $b_{0}\left(b_{1}b_{2}-b_{0}b_{3}\right)>0$ (hence, $b_{0}>0$) are needed. As expected, these conditions prove to be equivalent to the ones obtained via Jury’s criteria—we omit the details to avoid redundant repetition.

#### 4.2.1. The Kelvin–Voigt Model

Although the focus of the present paper is on the hyperbolic-like case corresponding to τ>0, the above calculations are valid for τ=0, the Kelvin–Voigt subfamily as well. As a brief analysis of this case, (58) is trivially satisfied with $\hat{\tau }>0$. (59) gives the nontrivial condition α<1/2. (Together with boundary conditions, this may be weakened to $\alpha \leq \frac{1}{2}$.) Finally, (60) gives

(64) $$\begin{array}{c} \left(\frac{1}{2}-\alpha \right)\Delta t^{2}+\hat{\tau }\Delta t<\frac{\frac{1}{2}-\alpha }{c^{2}}\Delta x^{2}, \end{array}$$

which looks like some mixture of a stability condition for a scheme for a parabolic problem, such as Fourier heat conduction, and of a condition for a simple reversible wave propagation.

#### 4.2.2. Beyond Kelvin–Voigt

When τ>0, then

(65) $$\begin{array}{c} \hat{C}=\hat{c}\frac{\Delta t}{\Delta x}>C \end{array}$$

becomes important (recall (14)).

The most interesting case is α=1/2, where the scheme yields second-order accurate predictions: (59) holds trivially, and (60) can be rewritten as

(66) $$\begin{array}{c} \hat{C}<1. \end{array}$$

With boundary conditions also present, we may extend this condition to

(67) $$\begin{array}{c} \hat{C}\leq 1. \end{array}$$

We considered the two other potentially interesting cases as well. If α=1, then (59) induces Δt<2τ, which is not a harsh requirement since the time step must usually be much smaller than the time scales of the system in order to obtain a physically acceptable numerical solution. In parallel, $\hat{C}$ is limited from above by a number smaller than 1. On the other side, when α=0, then (59) is automatically true again, and now $\hat{C}$ is limited from above by a number larger than 1. Since we may need Δt≪τ for a satisfactory solution, this $O\left(\frac{\Delta t}{\tau }\right)$ gain over 1 is not considerable.

#### 4.2.3. Hooke Case

It is worth looking back to the Hooke limit of (60): $\tau =\hat{\tau }=0$ (with whatever α) tells C<1. One can see that the $\left|\xi \right|<1$ stability requirement yields conservative results and does not tell us how far the obtained inequalities are from equalities.

## 5. Numerical Results

**Figure 2:**
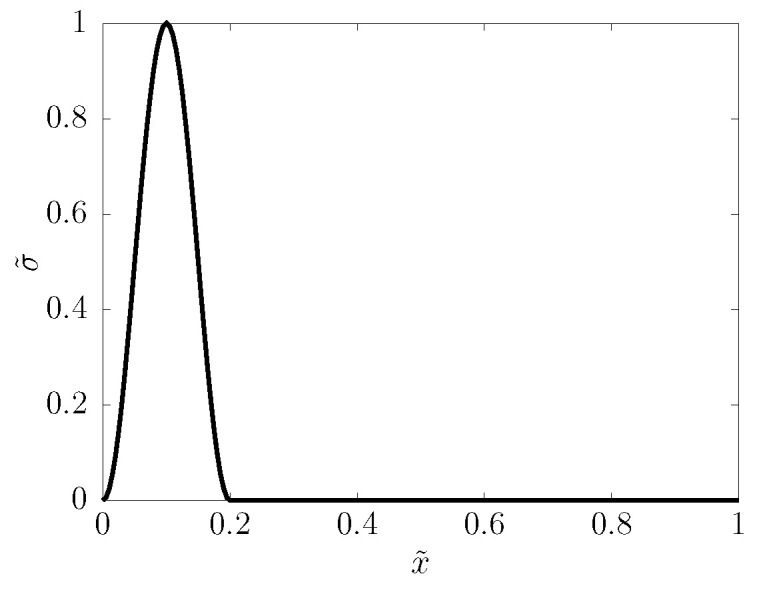
Snapshot of the shape of the fully born stress pulse near the left end of the sample.

The calculations communicated here are carried out with zero v,ε,σ as initial conditions, and with stress boundary conditions: on one end of the sample, a cosine-shaped pulse is applied (see Figure 2), while the other end is free (stress is zero). With $\tau_{b}$ denoting the temporal width of the pulse, the excitation is, hence,

(68) $$\begin{array}{c} \sigma \left(t,0\right)=\left\{\begin{array}{cc} \frac{\sigma_{b}}{2}\left[1-\cos\left(2\pi \frac{t}{\tau_{b}}\right)\right]\; & \mathrm{if}\;0\leq t\leq \tau_{b},\\ 0 & \mathrm{otherwise}. \end{array}\right. \end{array}$$

Temperature is calculated via the discretized form of (8), with the natural choice that temperature values reside at the same place as stress and strain, but half-shifted in time ($T_{n}^{j-1/2}$ at $t^{j}-\frac{\Delta t}{2},\;x_{n}$).

When plotting, say, elastic energy of the whole sample at time $t^{j}$, a simple $\frac{E}{2}\sum_{n}{\left(\varepsilon_{n}^{j}\right)}^{2}\Delta x$ type sum is used, with two adjustments. First, terms living at the outer endpoint of an outermost space cell, such as ${\left(\varepsilon_{0}^{j}\right)}^{2}$ and ${\left(\varepsilon_{N}^{j}\right)}^{2}$, are counted with weight $\frac{1}{2}$. Second, kinetic energy and thermal energy, both being based on quantities half-shifted in time, are calculated as a time average, their value at $t^{j}$ taken as the average of their value at $t^{j}-\frac{\Delta t}{2}$ and $t^{j}+\frac{\Delta t}{2}$.

The numerical calculations are performed for dimensionless quantities. For making the quantities dimensionless, the following units are used: the length of the sample *X*, *c* (so a Hookean wave arrives at the other end during unit time), *E*, $\sigma_{b}$, and $c_{\sigma }$. Accordingly, dimensionless position, time, velocity, stress, strain, energy, temperature, and wave number are defined as

(69) $$\begin{array}{ccccccccccccccc} \tilde{x} & =\frac{1}{X}x, & \tilde{t} & =\frac{c}{X}t, & \tilde{v} & =\frac{1}{c}v, & \tilde{\sigma } & =\frac{1}{\sigma_{b}}\sigma , & \tilde{\varepsilon } & =\frac{E}{\sigma_{b}}\varepsilon , & \tilde{e} & =\frac{E^{2}}{c^{2}\sigma_{b}^{2}}e, & \tilde{T} & =\frac{E^{2}c_{\sigma }}{c^{2}\sigma_{b}^{2}}T, & \tilde{k}=Xk. \end{array}$$

The results are presented for dimensionless time constants

(70) $$\begin{array}{ccccccccc} \; & \; & {\tilde{\tau }}_{b} & =0.2, & \tilde{\tau } & =1.25, & \tilde{\hat{\tau }} & =5,\; & \; \end{array}$$

the latter two implying $\hat{c}/c=2$.

### 5.1. Hookean Wave Propagation

**Figure 3:**
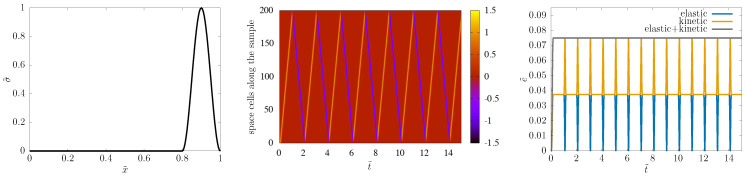
*Left:* Snapshot of the stress pulse right before its 15th bouncing back from the boundary. *Middle:* Spacetime picture of the wave propagation. Bouncing back from free ends makes stress change sign. *Right:* Elastic energy, kinetic energy, and their sum as functions of time. Calculation done with N=200 space cells and C=1.

For the Hooke system, our scheme is symplectic, with very reliable long-time behavior. This is well visible in Figure 3: the shape is nicely preserved, no numerical artefacts are visible in the spacetime picture, and the sum of elastic and kinetic energy is conserved.

### 5.2. Poynting–Thomson–Zener Wave Propagation

**Figure 4:**
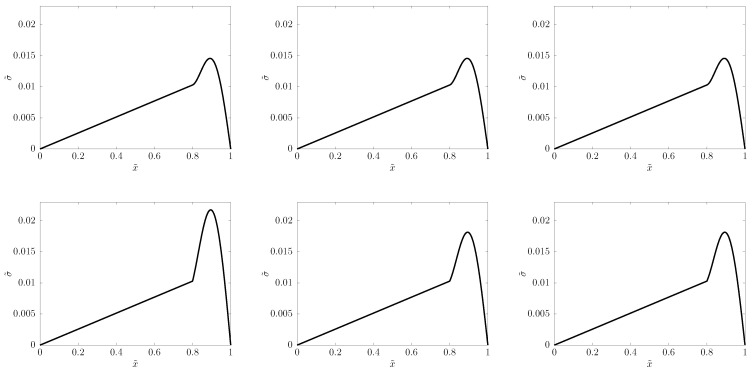
*Upper row:*α=1/2; *Lower row:*α=0—calculation of the stress signal when it starts its 7th bouncing, with $\hat{C}=1$. *From left to right:*N=400,800,1600 space cells.

For the PTZ system, we find that the principally optimal choice of α=1/2 does outperform α=0 (with $\hat{C}=1$). Figure 4 shows such a comparison: α=1/2 produces a reliable signal shape quite independently of space resolution, while α=0 needs more than N=1000 space cells to reach the same reliability.

**Figure 5:**
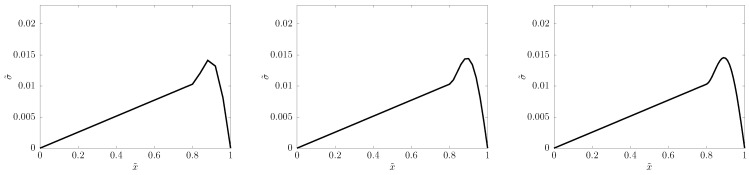
The same α=1/2 prediction with N=25,50,100 space cells, from left to right, respectively.

α=1/2 suggests that realibility already at N=50, and even N=25, “does a decent job,” as depicted in Figure 5.

**Figure 6:**
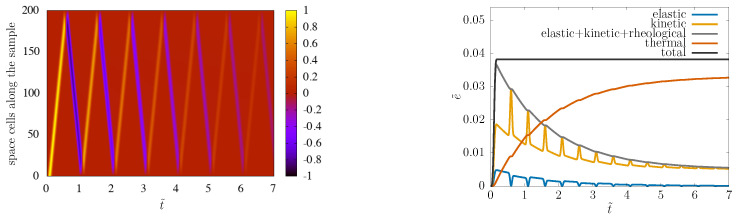
α=1/2, $\hat{C}=1$ spacetime picture and energy conservation, N=200.

With α=1/2, the spacetime picture and total energy conservation are not less satisfactory, as visible in Figure 6.

The physical explanation of the signal shape (Figure 4 and Figure 5) is that the fastest modes propagate with speed $\hat{c}$ (recall Section 2), transporting the front of the signal, while slow modes travel with $c<\hat{c}$, gradually falling behind, and forming, little by little, a thickening tail.

In parallel, the spacetime picture shows that this tail effect is less relevant than the overall decrease of the signal, due to dissipation.

Finally, concerning the energy results, the remarkable fact is that all ingredients v,ε,σ,T are calculated via discretized time integration, so total energy conservation is not built in, but is a test of the quality of the whole numerical approach. The observed good energy conservation behavior would deserve deeper analysis in the future, possibly analogously to [6].

## 6. Dissipation Error and Dispersion Error

### 6.1. Hooke Case

**Figure 7:**
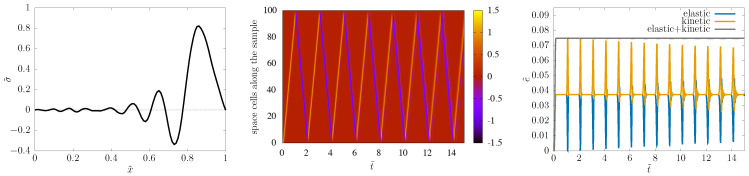
Wavy dispersion error and decrease by dissipative error for the Hooke system when C=1/2, with N=100.

**Figure 8:**
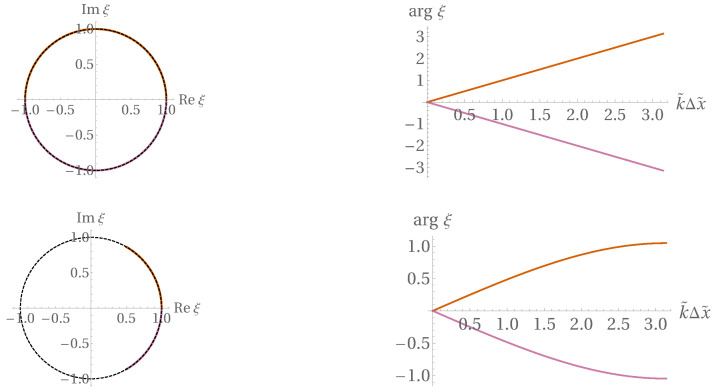
*Upper row:* Case of C=1; *lower row:* case of C=1/2. *Left:* The two roots $\xi_{\pm }$ in the complex plane; *right:* *k* dependence of the argument of $\xi_{\pm }$.

The Hooke system might appear as a simple introductory task for numerics. This is actually far from true. The Hooke case already displays both dissipation error and dispersion error if not treated with appropriate care (see Section 7 below, as well as [28]). While the greatest danger, instability, is about an exponential exploding of error, dissipation error is “the opposite”: when the signal decreases in time, losing energy due to numerical artefact only. This type of error is related to $\left|\xi \right|<1$ modes, which indicates that one should try to stay on the unit circle with ξ. On the other side, in addition to the modulus of ξ, its argument can also cause trouble: if argξ is not linear in *k*, then dispersion error is induced, which is observable as unphysical waves generated numerically around signal fronts. These errors are present even in a symplectic scheme such as ours, as illustrated in Figure 7. More insight is provided by Figure 8.

### 6.2. Poynting–Thomson–Zener Case

**Figure 9:**
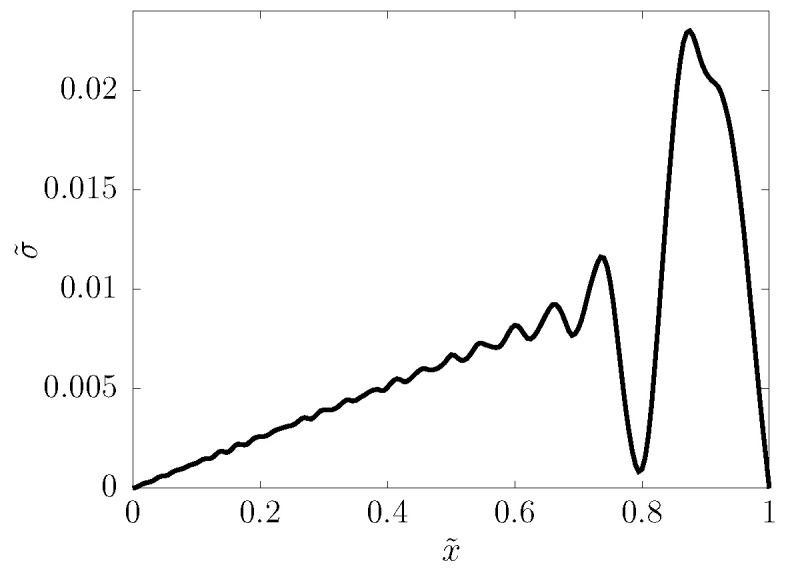
The stress signal provided by the scheme with $\hat{C}=1/2$, N=200, for comparison with Figure 5. (All other settings are the same.)

In case of a dissipative system such as the PTZ one, it is hard to detect the dissipative error, i.e., to distinguish it from the physical dissipation. The dispersion error remains visible, as Figure 9 shows.

Usually, one would need to set Δt to be much smaller than τ, $\hat{\tau }$ (and $\tau_{b}$) to obtain a physically acceptable approximation. Rewriting the coefficients (53) as

(71) $$\begin{array}{cc} a_{0} & =\frac{\alpha \frac{\Delta t}{\tau }-1}{\left(1-\alpha \right)\frac{\Delta t}{\tau }+1}=-1+O\left(\frac{\Delta t}{\tau }\right),\;a_{1}=3-\frac{2\frac{\Delta t}{\tau }-4\left(\alpha \frac{\Delta t}{\tau }-\frac{\hat{\tau }}{\tau }\right)C^{2}S^{2}}{\left(1-\alpha \right)\frac{\Delta t}{\tau }+1}=3-4{\hat{C}}^{2}S^{2}+O\left(\frac{\Delta t}{\tau }\right),\\ a_{2} & =-3+\frac{\frac{\Delta t}{\tau }+4\left[\left(1-\alpha \right)\frac{\Delta t}{\tau }+\frac{\hat{\tau }}{\tau }\right]C^{2}S^{2}}{\left(1-\alpha \right)\frac{\Delta t}{\tau }+1}=-3+4{\hat{C}}^{2}S^{2}+O\left(\frac{\Delta t}{\tau }\right),\;a_{3}=1, \end{array}$$

in the limit $\frac{\Delta t}{\tau }\to 0$, the characteristic polynomial reduces to

(72) $$\begin{array}{c} \xi^{3}+\left(-3+4{\hat{C}}^{2}S^{2}\right)\xi^{2}+\left(3-4{\hat{C}}^{2}S^{2}\right)\xi -1=\left(\xi -1\right)\left[\xi^{2}+\left(-2+4{\hat{C}}^{2}S^{2}\right)\xi +1\right], \end{array}$$

with roots satisfying $\xi_{0}=\left|\xi_{+}\right|=\left|\xi_{-}\right|=1$, excluding thus the dissipation error. Especially simple and distinguished is the case $\hat{C}=1$, when the roots are

(73) $$\begin{array}{c} \xi_{0}=1,\;\xi_{\pm }=e^{\pm ik\Delta x}, \end{array}$$

providing dispersion relations linear in *k* and, hence, getting rid of dispersion error as well.

**Figure 10:**
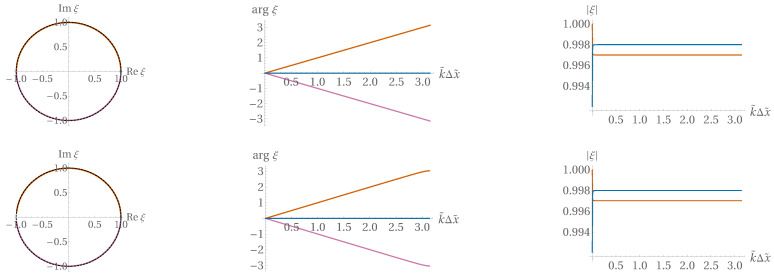
Visualization of the three branches $\xi_{0}\left(k\right),\xi_{+}\left(k\right),\xi_{-}\left(k\right)$ for $\hat{C}=1$. *Upper row:* α=1/2; *lower row:*α=0.

**Figure 11:**
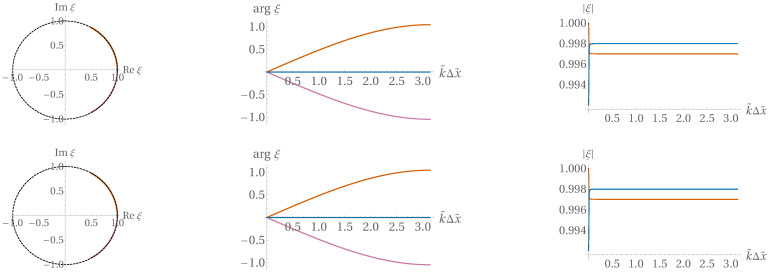
Same as Figure 10, but with $\hat{C}=1/2$.

**Figure 12:**
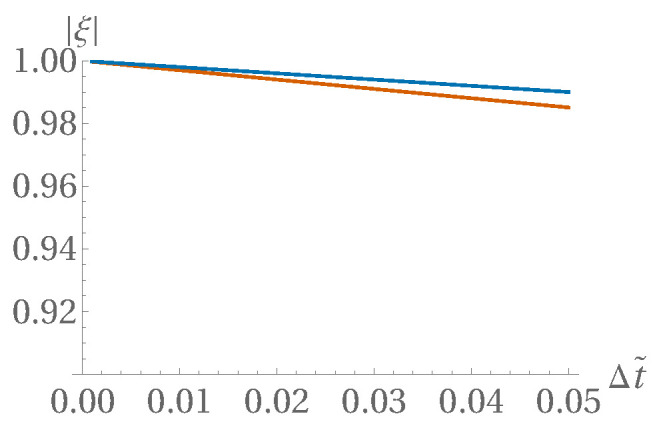
In Figure 10 and Figure 11, the roots are not exactly on the unit circle—here, Δt dependence of $\left|\xi_{0}\right|$ and $\left|\xi_{\pm }\right|$ is displayed, at a neutral value kΔx=π/4, for $\hat{C}=1$ and α=1/2.

With slightly nonzero $\frac{\Delta t}{\tau }$, these nice properties are detuned but only up to $O\left(\frac{\Delta t}{\tau }\right)$, as shown in Figure 10, Figure 11 and Figure 12 (prepared at a dimensionless time step value of 0.01; the detuning appears weaker for α=1/2 than for α=0).

## 7. Solutions Using the Finite Element Software COMSOL

Finally, for comparative reasons, we present solutions obtained via a commercial finite element software, namely, COMSOL v5.3a. We considered the Hookean case, for which the COMSOL implementation is straightforward since the built-in mathematical environment offers the possibility to solve such classical partial differential equations, too.

For the finite element realization, we chose the displacement field as the primary field variable. Velocity, plotted in the figures below, is then obtained by taking its time derivative.

To conform with the units used for defining the dimensionless quantities, we set both the propagation speed and the sample length to unity. The spatial domain consisted of 100 elements, obtained using the options of a “physics-controlled mesh” and an “extremely fine” element size. On the boundaries, the gradients were prescribed, and the excitation was given analogously to our above simulations (see (68) and Figure 2). We examined the schemes for two different pulse lengths, ${\tilde{\tau }}_{b}=0.2$ and ${\tilde{\tau }}_{b}=0.04$.

In what follows, we tested five different settings for time stepping, in order to find the appropriate ones and to compare their effectiveness. For the simulations, we used a configuration of an i7-7700 CPU with 3.6 GHz and 16 GB RAM. COMSOL supports parallel computing, which is an option that has been exploited. Although the run time strongly depends on other factors, it provides a good picture for comparing the effectiveness of the commercial approach and the scheme presented in this paper.

**Figure 13:**
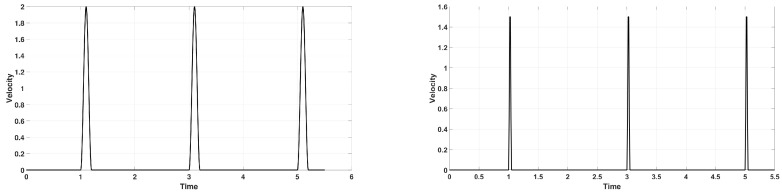
Applying the proposed scheme in the case of two different pulse lengths; ${\tilde{\tau }}_{b}=0.2$ (**left**) and ${\tilde{\tau }}_{b}=0.04$ (**right**). The dimensionless space and time steps are $\Delta \tilde{x}=\Delta \tilde{t}=0.01$. This time step is actually not much smaller than the shorter pulse length, so, for example, the tips cannot be plotted accurately when ${\tilde{\tau }}_{b}=0.04$, but the solution still performs well.

Our scheme, using the same number of spatial elements and time interval, ran in around 0.2 s in Matlab (using 1 core only) for both pulse lengths, as measured by Matlab. First, we present the results of our scheme (see Figure 13). The solutions are apparently free of dissipation and of dispersion.

Next, we present the outputs obtained via COMSOL used with various settings.

### 7.1. Backward Differentiation Formula (BDF), Order 2 and Order 5

**Figure 14:**
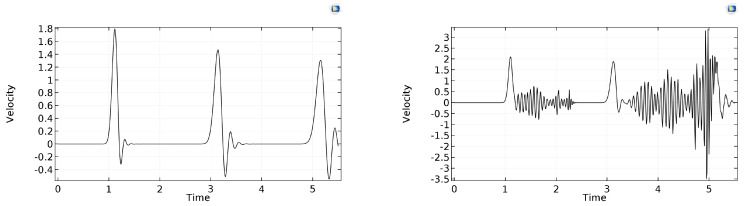
Rear-side velocity history in time for ${\tilde{\tau }}_{b}=0.2$, with maximum BDF order being 2 (**left**) and 5 (**right**), respectively.

In its simplest version, it is the backward Euler scheme that has good stability properties, with artificial damping effects. As shown by the comparison in Figure 14, artificial damping is stronger for the lower-order version (i.e., maximum BDF order is 2), while, with higher-order schemes, the damping is less significant; therefore, the artificial oscillations are less suppressed. The run time is between 30–40 s.

### 7.2. Runge–Kutta-Based Schemes: Cash–Karp 5

This scheme results in unstable solutions, independently of the corresponding settings (initial time step, time step control, and stiffness control).

### 7.3. Runge–Kutta-Based Schemes: Dormand–Prince 5

**Figure 15:**
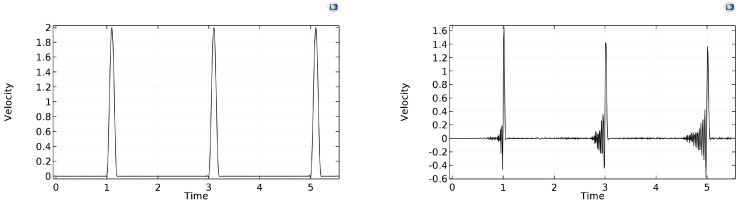
Rear-side velocity history in time, using the Dormand–Prince time stepping method (**left**: ${\tilde{\tau }}_{b}=0.2$; **right**: ${\tilde{\tau }}_{b}=0.04$).

With this scheme, the numerical stability of the solution strongly depends on the settings of the maximum step size growth ratio and the step size safety factor. At default settings, the solution is unstable. Using 0.1 for the step size safety factor and 0.01 for the maximum step size, the results can be seen in Figure 15. Only small oscillations are observable at the wave front; however, the computation requires almost 2 GB RAM. The run time is strongly influenced by the pulse length. For ${\tilde{\tau }}_{b}=0.2$, it runs at around 300–320 s, using all available computing capacity. Meanwhile, for the shorter pulse length ${\tilde{\tau }}_{b}=0.04$, it needs more than 580 s. In addition, for smaller pulse length, dispersive and dissipative errors are also visible.

### 7.4. Runge–Kutta-Based Schemes: RK34

**Figure 16:**
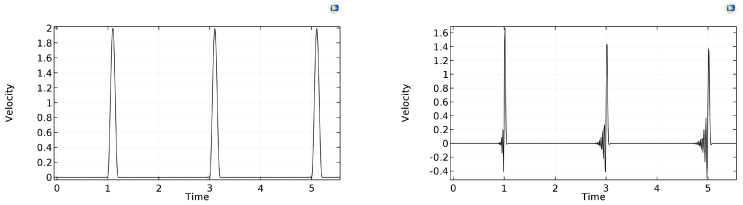
Rear-side velocity history in time, using the RK34 time stepping method (**left**: ${\tilde{\tau }}_{b}=0.2$, **right**: ${\tilde{\tau }}_{b}=0.04$).

Using stiffness detection, this scheme solves the problem in the fastest and most efficient way. However, when the pulse length is ${\tilde{\tau }}_{b}=0.04$, then both its damping and dispersive properties become apparent (see Figure 16). With this method, the run time was around 45 s.

**Figure 17:**
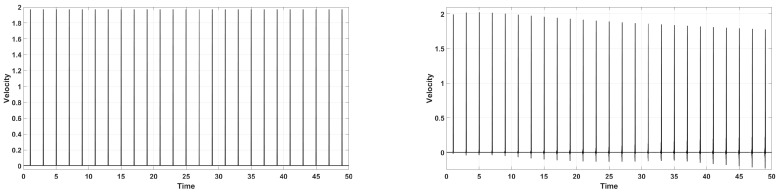
Rear-side velocity history in time, for pulse length ${\tilde{\tau }}_{b}=0.04$, with 300 nodes. (**Left**) solution by our scheme; (**right**) COMSOL RK34 result.

Since this COMSOL option proved the best, in order to test the mesh dependence of its solution, we examined the ${\tilde{\tau }}_{b}=0.04$ case with 300 space cells ($\Delta \tilde{x}=\Delta \tilde{t}=0.0033$) as well, for a longer process (100 bounces). With these settings, our scheme required 0.3 s in Matlab and shows no numerical artefact, while the COMSOL solution took 9649 s and exhibits apparent dissipative error and mild but increasing dispersion error around the rear of the pulse (see Figure 17).

To summarize, compared to our scheme realized in Matlab, COMSOL run times are 100–1000–10,000 times larger, with large memory demand, and various settings have to be tuned to obtain a stable solution with moderate artificial dissipation and dispersion.

## 8. Discussion

Choosing a good finite difference numerical scheme for a continuum thermodynamical problem is not easy. A good starting point can be a symplectic scheme for the reversible part, as done here, too. Another advantage is provided by a staggered arrangement of quantities by half space and time steps, suited to balances, to the kinematic equations, to the Onsagerian equations, etc.

Even with all such preparations, instability is a key property to ensure. When all these are settled, dissipative and dispersive errors can invalidate our calculation, which may not be recognized when the continuum system is dissipative or when it allows wave propagation.

Notably, there is a principal difference between the stability problems of a numerical method and the stability issues for a continuum phenomenon itself. The former are induced by the approximations and depend on the type of approximation, while the original continuum system may be fine regarding stability—for example, ensured by thermodynamical consistence. It is interesting to realize that, nevertheless, these two types of instability are not completely independent. In one of the directions, the stability investigation of a numerical scheme may provide information for the underlying continuum phenomenon as well. An example for this has been provided by our (58) above, which is a condition that is independent of the time step, the space step, the parameter α that parametrizes the scheme, and any other aspect of the scheme. Rather, it is a condition on the underlying continuum model. In the present case, we already know this condition as one of the stability requirements imposed by thermodynamical consistency, seen at (9). This example illustrates that, in more complicated problems, it is also worth investigating the stability conditions of the numerical method and trying to distill *scheme-independent information* on the continuum system from them.

In parallel, the other direction is when the stability of a continuum system can be used *to devise stable numerical methods*. One such example is the case of symplectic numerical schemes, which are actually exact integrators of a certain Hamiltonian system—a slightly different one from the original system. The generalization of this way of thinking to nonconservative systems is a promising research direction.

Concerning the future prospects of the study provided here, the findings can be supplemented by comparison with analytical solutions and further finite element calculations, performed for the whole PTZ system.

Another logical continuation of the present line of research is extension of the scheme to 2D and 3D space—this is actually a work in progress [28].

Regarding the thermodynamical system to be investigated, the whole Kluitenberg–Verhás family—which the present PTZ model is a subclass of—could be studied. The presence of the second derivative of strain, and actually already the Kelvin–Voigt subfamily, brings in the aspect of parabolic characteristics, so useful implications may be gained for other thermodynamical areas such as non-Fourier heat conduction.

Reliable numerical methods for thermodynamical systems, which avoid all the various pitfalls, are an important direction for future research.
